# Supplementary material for: High-Risk Genotypes of Human Papillomavirus at Diverse Anogenital Sites among Chinese Women: Infection Features and Potential Correlation with Cervical Intraepithelial Neoplasia
Source: Cancers (Basel). 2024 May 31;16(11):2107. doi: 10.3390/cancers16112107 (PMC11172096; doi:10.3390/cancers16112107)
Supplement: Supplementary file 1 [file cancers-16-02107-s001.zip › cancers-2984210-supplementary.pdf]

High-risk genotypes of human papillomavirus at diverse anogenital sites among Chinese women: infection features and potential correlation with cervical intraepithelial neoplasia

Chao Zhao [1,†], Jiahui An [2,3,†], Mingzhu Li [1], Jingran Li [1], Yun Zhao [1], Jianliu Wang [1], Heidi Qunhui Xie [2,3,*] and Lihui Wei [1,*]

1 Department of Obstetrics and Gynecology, Peking University People's Hospital, Beijing 100044, China; zhaochaormyy@bjmu.edu.cn (C.Z.); mingzhu1815@bjmu.edu.cn (M.L.); lijingran@pkuph.edu.cn (J.L.); zhaoyun@pkuph.edu.cn (Y.Z.); wangjianliu@pkuph.edu.cn (J.W.)
2 State Key Laboratory of Environmental Chemistry and Ecotoxicology, Research Center for Eco-Environmental Sciences, Chinese Academy of Sciences, Beijing 100085, China; jhan2022_st@rcees.ac.cn
3 University of Chinese Academy of Sciences, Beijing 100049, China
* Correspondence: qhxie@rcees.ac.cn (H.Q.X.); weilh@bjmu.edu.cn (L.W.)
† These authors contributed equally to this work.

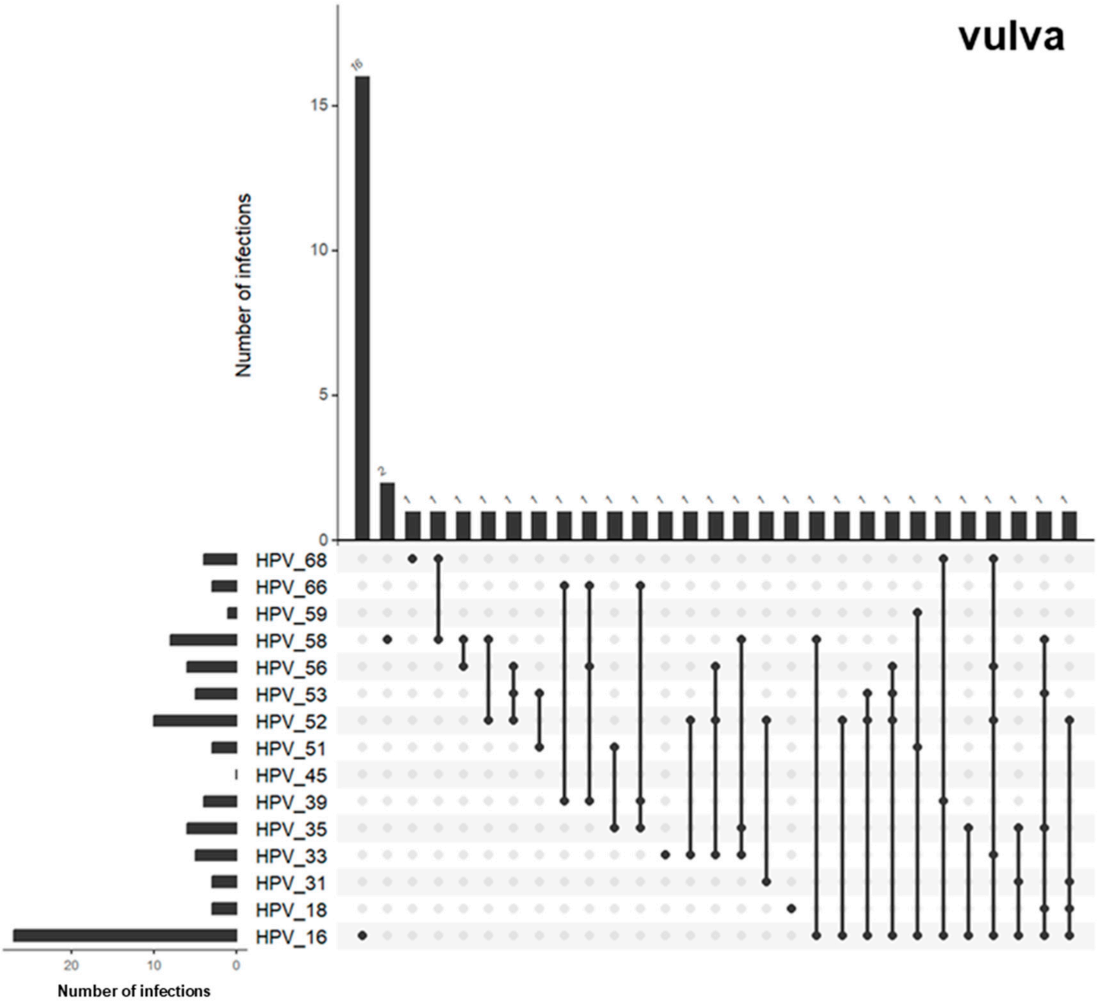

Figure S1. Venn plot shows the prevalence of both single- and muti-infection patterns in vulva for patients with cervical intraepithelial lesions (CIN) 2+ (n=45) based on UpSet results. The total prevalence of each HR-HPV is shown on the left, while the number of each infection pattern is presented at the top.

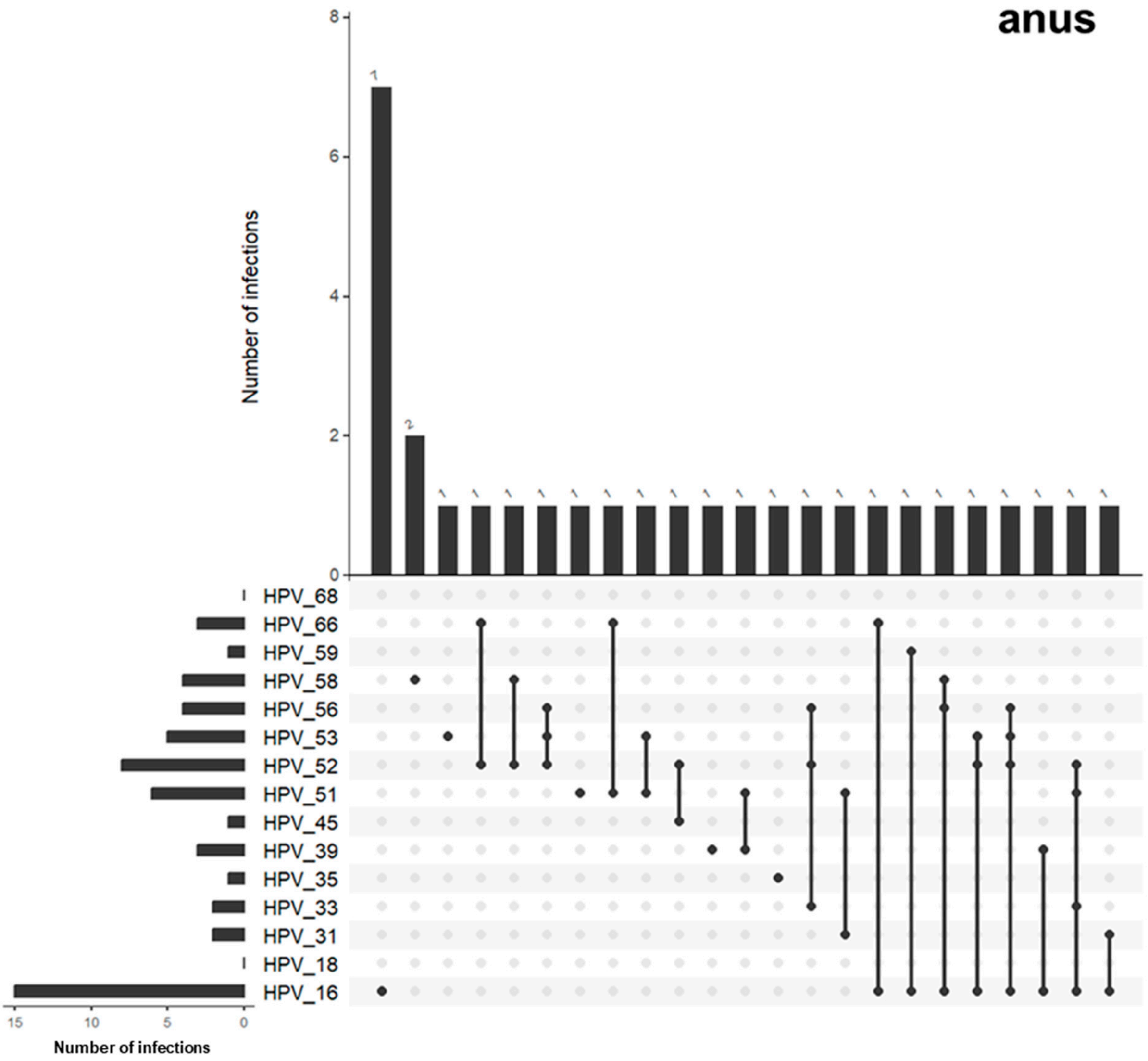

Figure S2. Venn plot shows the prevalence of both single- and muti-infection patterns in anus for patients with CIN 2+ based on UpSet results (*n*=30). The total HPV prevalence of each HR-HPV is shown on the left, while the number of each infection pattern is presented at the top.

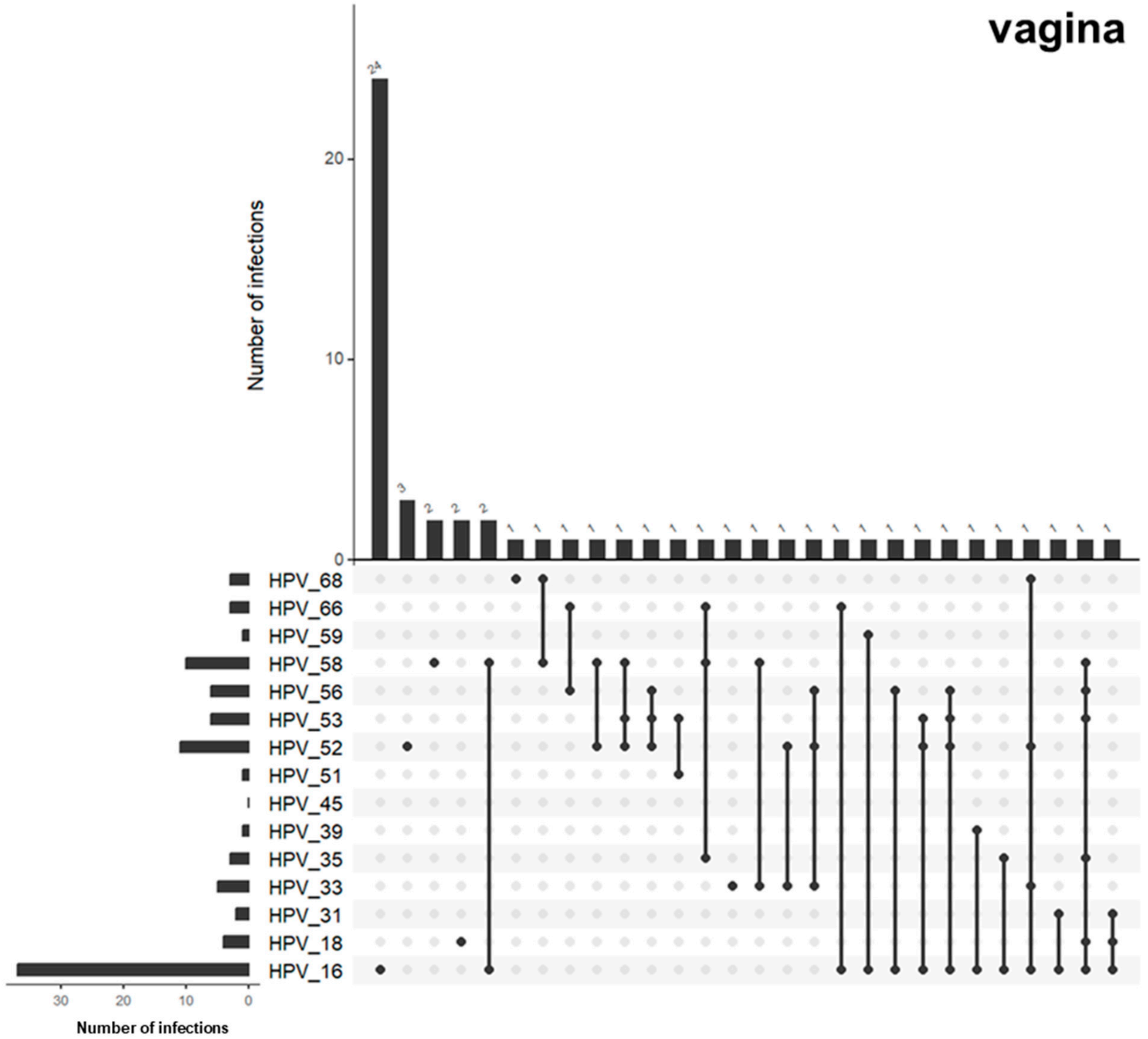

Figure S3. Venn plot shows the prevalence of both single- and muti-infection patterns in vagina for patients with CIN 2+ based on UpSet results (*n*=56). The total HPV prevalence of each HR-HPV is shown on the left, while the number of each infection pattern is presented at the top.

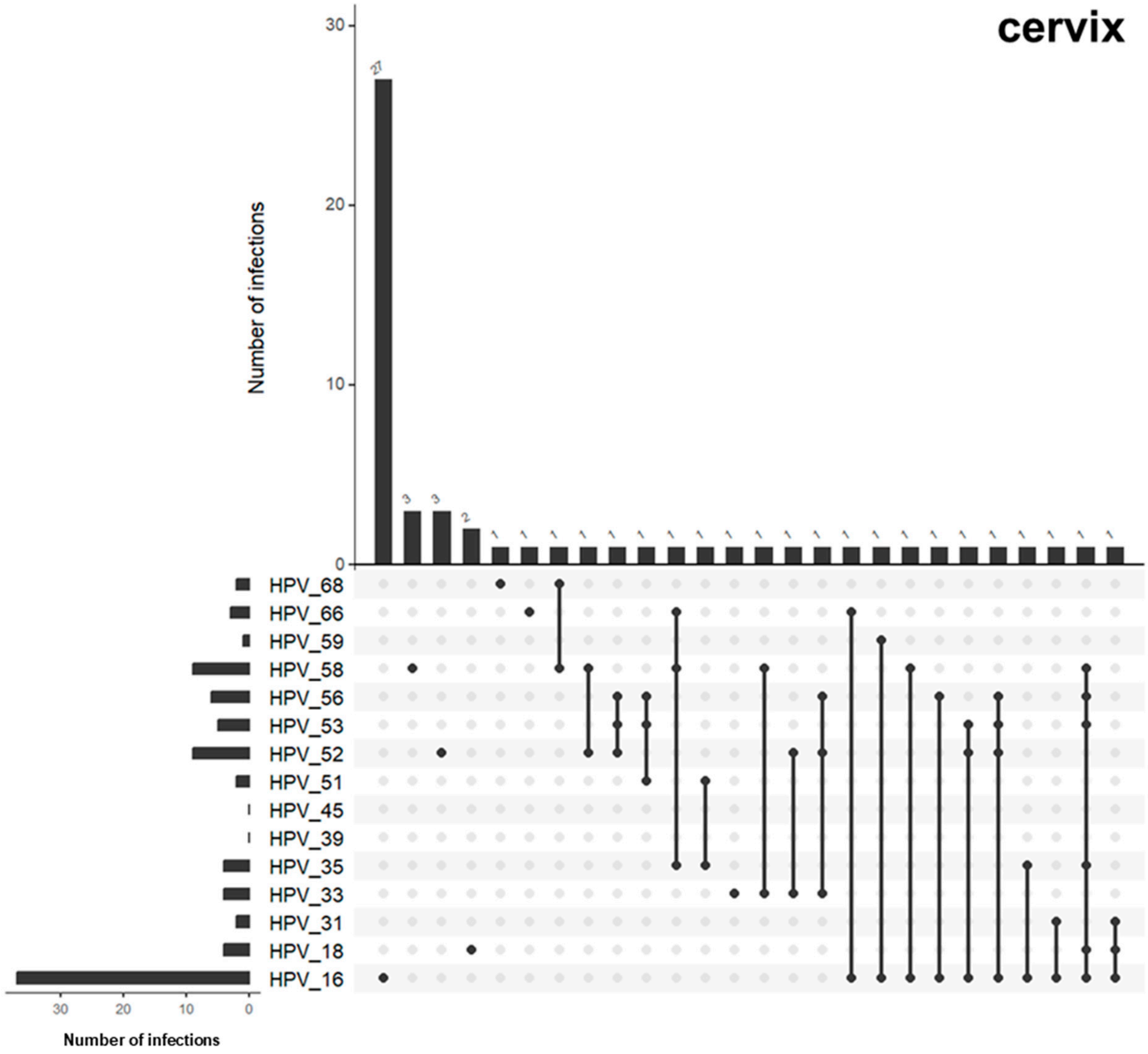

Figure S4. Venn plot shows the prevalence of both single- and muti-infection patterns in cervix for patients with CIN 2+ based on UpSet results (*n*=57). The total HPV prevalence of each HR-HPV is shown on the left, while the number of each infection pattern is presented at the top.

Table S1. Features of single/ multiple infection at the four anatomical sites.

Infection pattern		Anatomical site			
		Vulva	Anus	Vagina	Cervix
**single infection**	counts[1]	138	94	215	230
	proportion[2]	46.3%[a]	47.7%[a]	61.1%[b]	63.5%[b]
**double infection**	counts	91	66	83	88
	proportion	30.5%[a]	33.5%[a]	23.6%[a]	24.3%[a]
**triple infection**	counts	51	27	34	27
	proportion	17.1%[a]	13.7%[a, b]	9.7%[b]	7.5%[b]
**quadruple infection**	counts	12	9	16	12
	proportion	4.0%[a]	4.6%[a]	4.5%[a]	3.3%[a]
**five-fold infection**	counts	6	1	3	4
	proportion	2.0%[a]	0.5%[a]	0.9%[a]	1.1%[a]
**six-fold infection**	counts	0	0	1	1
	proportion	0.0%[a]	0.0%[a]	0.3%[a]	0.3%[a]
**total**	counts	298	197	352	362
	proportion	100.0%	100.0%	100.0%	100.0%

[1] count: the number of cases of each infection type at four anatomical sites.

[2] proportion: the percentage of each infection pattern cases to the total cases at one anatomical site.

[a, b] For all proportions with the same letter, the difference between two anatomical sites is not statistically significant. If two proportions have different letters, they are significantly different.

Table S2. Comparison of single and multiple infection of 15 HR-HPVs in vulva.

HPV subtypes		Type of infection		Total
		Single infection	Multiple infection	
HPV 16	counts[1]	51	49	100
	proportion[2]	51.0%$_a$	49.0%$_b$	100.0%
HPV 18	counts	4	8	12
	proportion	33.3%$_a$	66.7%$_a$	100.0%
HPV 31	counts	3	15	18
	proportion	16.7%$_a$	83.3%$_a$	100.0%
HPV 33	counts	5	17	22
	proportion	22.7%$_a$	77.3%$_a$	100.0%
HPV 35	counts	0	12	12
	proportion	0.0%$_a$	100.0%$_b$	100.0%
HPV 39	counts	2	27	29
	proportion	6.9%$_a$	93.1%$_b$	100.0%
HPV 45	counts	1	2	3
	proportion	33.3%$_a$	66.7%$_a$	100.0%
HPV 51	counts	6	52	58
	proportion	10.3%$_a$	89.7%$_b$	100.0%
HPV 52	counts	20	69	89
	proportion	22.5%$_a$	77.5%$_a$	100.0%
HPV 53	counts	11	45	56
	proportion	19.6%$_a$	80.4%$_a$	100.0%
HPV 56	counts	11	35	46
	proportion	23.9%$_a$	76.1%$_a$	100.0%
HPV 58	counts	12	36	48
	proportion	25.0%$_a$	75.0%$_a$	100.0%
HPV 59	counts	1	12	13
	proportion	7.7%$_a$	92.3%$_a$	100.0%
HPV 66	counts	6	18	24
	proportion	25.0%$_a$	75.0%$_a$	100.0%
HPV 68	counts	5	16	21
	proportion	23.8%$_a$	76.2%$_a$	100.0%

[1] count: the number of single or multiple infection of each HR-HPV subtype in vulva.

[2] proportion: the percentage of single or multiple infection cases to the total cases regarding each HR-HPV subtype in vulva.

[a, b] For all proportions with the same letter, the difference of each HPV subtype between single infection and multiple infection is not statistically significant. If two proportions have different letters, they are significantly different.

Table S3. Comparison of single and multiple infection of 15 HR-HPVs in anus.

HPV Subtypes		Type of infection		Total
		Single infection	Multiple infection	
HPV 16	counts[1]	30	29	59
	proportion[2]	50.8%$_a$	49.2%$_b$	100.0%
HPV 18	counts	2	0	2
	proportion	100.0%$_a$	0.0%$_b$	100.0%
HPV 31	counts	2	10	12
	proportion	16.7%$_a$	83.3%$_a$	100.0%
HPV 33	counts	0	13	13
	proportion	0.0%$_a$	100.0%$_b$	100.0%
HPV 35	counts	1	2	3
	proportion	33.3%$_a$	66.7%$_a$	100.0%
HPV 39	counts	4	19	23
	proportion	17.4%$_a$	82.6%$_a$	100.0%
HPV 45	counts	1	2	3
	proportion	33.3%$_a$	66.7%$_a$	100.0%
HPV 51	counts	7	37	44
	proportion	15.9%$_a$	84.1%$_a$	100.0%
HPV 52	counts	18	51	69
	proportion	26.1%$_a$	73.9%$_a$	100.0%
HPV 53	counts	6	26	32
	proportion	18.8%$_a$	81.3%$_a$	100.0%
HPV 56	counts	6	21	27
	proportion	22.2%$_a$	77.8%$_a$	100.0%
HPV 58	counts	7	16	23
	proportion	30.4%$_a$	69.6%$_a$	100.0%
HPV 59	counts	1	7	8
	proportion	12.5%$_a$	87.5%$_a$	100.0%
HPV 66	counts	6	13	19
	proportion	31.6%$_a$	68.4%$_a$	100.0%
HPV 68	counts	3	8	11
	proportion	27.3%$_a$	72.7%$_a$	100.0%

[1] count: the number of single or multiple infection of each HR-HPV subtype in anus.

[2] proportion: the percentage of single or multiple infection cases to the total cases regarding each HR-HPV subtype in anus.

[a, b] For all proportions with the same letter, the difference of each HPV subtype between single infection and multiple infection is not statistically significant. If two proportions have different letters, they are significantly different.

Table S4. Comparison of 15 HR-HPVs between single and multiple infection in vagina.

HPV Subtypes		Type of infection		Total
		Single infection	Multiple infection	
HPV 16	counts[1]	75	47	122
	proportion[2]	61.5%a	38.5%b	100.0%
HPV 18	counts	15	8	23
	proportion	65.2%a	34.8%b	100.0%
HPV 31	counts	4	12	16
	proportion	25.0%a	75.0%a	100.0%
HPV 33	counts	4	17	21
	proportion	19.0%a	81.0%a	100.0%
HPV 35	counts	1	9	10
	proportion	10.0%a	90.0%a	100.0%
HPV 39	counts	8	18	26
	proportion	30.8%a	69.2%a	100.0%
HPV 45	counts	1	2	3
	proportion	33.3%a	66.7%a	100.0%
HPV 51	counts	7	41	48
	proportion	14.6%a	85.4%b	100.0%
HPV 52	counts	30	52	82
	proportion	36.6%a	63.4%a	100.0%
HPV 53	counts	14	41	55
	proportion	25.5%a	74.5%b	100.0%
HPV 56	counts	15	29	44
	proportion	34.1%a	65.9%a	100.0%
HPV 58	counts	23	34	57
	proportion	40.4%a	59.6%a	100.0%
HPV 59	counts	6	9	15
	proportion	40.0%a	60.0%a	100.0%
HPV 66	counts	4	22	26
	proportion	15.4%a	84.6%b	100.0%
HPV 68	counts	8	12	20
	proportion	40.0%a	60.0%a	100.0%

[1] count: the number of single or multiple infection of each HR-HPV subtype in vagina.

[2] proportion: the percentage of single or multiple infection cases to the total cases regarding each HR-HPV subtype in vagina.

a, b For all proportions with the same letter, the difference of each HPV subtype between single infection and multiple infection is not statistically significant. If two proportions have different letters, they are significantly different.

Table S5. Comparison of single and multiple infection of 15 HR-HPVs in cervix.

HPV Subtypes		Type of infection		Total
		Single infection	Multiple infection	
HPV 16	counts[1]	75	43	118
	proportion[2]	63.6%a	36.4%b	100.0%
HPV 18	counts	18	15	33
	proportion	54.5%a	45.5%a	100.0%
HPV 31	counts	3	15	18
	proportion	16.7%a	83.3%b	100.0%
HPV 33	counts	6	16	22
	proportion	27.3%a	72.7%a	100.0%
HPV 35	counts	2	12	14
	proportion	14.3%a	85.7%b	100.0%
HPV 39	counts	7	15	22
	proportion	31.8%a	68.2%a	100.0%
HPV 45	counts	1	0	1
	proportion	100.0%a	0.0%a	100.0%
HPV 51	counts	9	31	40
	proportion	22.5%a	77.5%b	100.0%
HPV 52	counts	34	54	88
	proportion	38.6%a	61.4%a	100.0%
HPV 53	counts	16	32	48
	proportion	33.3%a	66.7%a	100.0%
HPV 56	counts	17	30	47
	proportion	36.2%a	63.8%a	100.0%
HPV 58	counts	24	31	55
	proportion	43.6%a	56.4%a	100.0%
HPV 59	counts	4	9	13
	proportion	30.8%a	69.2%a	100.0%
HPV 66	counts	7	17	24
	proportion	29.2%a	70.8%a	100.0%
HPV 68	counts	7	11	18
	proportion	38.9%a	61.1%a	100.0%

[1] count: the number of single or multiple infection of each HR-HPV subtype in anus.

[2] proportion: the percentage of single or multiple infection cases to the total cases regarding each HR-HPV subtype in cervix.

a, b For all proportions with the same letter, the difference of each HPV subtype between single infection and multiple infection is not statistically significant. If two proportions have different letters, they are significantly different.

Table S6. The infection distribution of 8 selected HR-HPVs at different anogenital sites of patients with CIN 2+.

Anogenital sites	Selected HR-HPVs							
	HPV 16	HPV 18	HPV 33	HPV 35	HPV 52	HPV 53	HPV 56	HPV 58
Cervix-included								
Cervix	0	0	0	0	0	0	1	0
Cervix+Vagina	8	1	0	0	1	0	2	1
Cervix+Vulva	0	0	0	1	0	0	0	0
Cervix+Anus	0	0	0	0	0	0	0	0
Cervix+Vagina+Vulva	15	3	2	2	2	1	0	4
Cervix+Vagina+Anus	2	0	0	0	1	0	0	0
Cervix+Vulva+Anus	0	0	0	0	0	0	0	0
Cervix+Vagina+Vulva+Anus	12	0	2	1	5	4	3	4
Cervix-excluded								
Vagina	0	0	0	0	1	1	0	1
Vulva	0	0	0	2	2	0	2	0
Anus	1	0	0	0	2	1	1	0
Vagina+Vulva	0	0	1	0	1	0	1	0
Vagina+Anus	0	0	0	0	0	0	0	0
Vulva+Anus	0	0	0	0	0	0	0	0
Vagina+Vulva+Anus	0	0	0	0	0	0	0	0
Total[1]	38	4	5	6	15	7	10	10

[1]Total: means the total number of cases of infection with a specific HR-HPV subtype in CIN 2+ patients.

Table S7. AUC values of different HR-HPVs muti-infection combinations detected in cervix for patients with CIN 2+.

Tests	AUC value[1]
HPV 16/33/58	0.753
HPV 16/33/35	0.753
HPV 16/33/35/58	0.752
HPV 16/18/33/58	0.750
HPV 16/18/33/35/58	0.748
HPV 16/18/33/35	0.747
HPV 16/33	0.746
HPV 16/58	0.742
HPV 16/35/58	0.741
HPV 16/18/33	0.740
HPV 16/33/35/53/58	0.739
HPV 16/18/58	0.739
HPV 16/33/35/53	0.738
HPV 16/33/53/58	0.738
HPV 16/18/33/35/53/58	0.737
HPV 16/18/35/58	0.737
HPV 16/18/33/53/58	0.736
HPV 16/35	0.735
HPV 16/33/35/56/58	0.735
HPV 16/33/56/58	0.734
HPV 16/18/33/35/53	0.734
HPV 16/18/33/35/56/58	0.733
HPV 16/18/33/56/58	0.732
HPV 16/33/35/56	0.731
HPV 16/35/56/58	0.731
HPV 16/56/58	0.731
HPV 16/18/35	0.730
HPV 16/18/35/56/58	0.729
HPV 16/18/56/58	0.729
HPV 16/18/33/35/56	0.728
HPV 16	0.728
HPV 16/33/53	0.728
HPV 16/35/53/58	0.726
HPV 16/35/52/58	0.724
HPV 16/53/58	0.724
HPV 16/18/35/53/58	0.724
HPV 16/18/33/53	0.724
HPV 16/33/35/52	0.723
HPV 16/18	0.723
HPV 16/18/35/52/58	0.722
HPV 16/18/53/58	0.722
HPV 16/33/56	0.722
HPV 16/52/58	0.721
HPV 16/33/35/52/58	0.720

HPV 16/35/56	0.720
HPV 16/18/33/35/52	0.720
HPV 16/35/52	0.720
HPV 16/18/52/58	0.719
HPV 16/18/33/56	0.719
HPV 16/18/33/35/52/58	0.718
HPV 16/33/52/58	0.717
HPV 16/35/53	0.717
HPV 16/18/35/56	0.717
HPV 16/18/35/52	0.717
HPV 16/18/33/52/58	0.715
HPV 16/18/35/53	0.713
HPV 16/33/52	0.712
HPV 16/56	0.711
HPV 16/18/33/52	0.709
HPV 16/52	0.708
HPV 16/18/56	0.708
HPV 16/33/35/53/56/58	0.708
HPV 16/53	0.707
HPV 16/18/33/35/53/56/58	0.707
HPV 16/35/52/53/58	0.706
HPV 16/35/52/56/58	0.706
HPV 16/18/52	0.705
HPV 16/18/35/52/53/58	0.705
HPV 16/18/35/52/56/58	0.705
HPV 16/33/35/52/53	0.705
HPV 16/33/35/52/53/58	0.704
HPV 16/33/35/52/56	0.704
HPV 16/33/53/56/58	0.704
HPV 16/18/33/35/52/53/58	0.703
HPV 16/33/35/52/56/58	0.703
HPV 16/35/53/56/58	0.703
HPV 16/52/56/58	0.703
HPV 16/18/33/35/52/53	0.703
HPV 16/18/33/53/56/58	0.703
HPV 16/18/53	0.703
HPV 16/18/33/35/52/56/58	0.702
HPV 16/18/35/53/56/58	0.702
HPV 16/33/35/53/56	0.702
HPV 16/18/52/56/58	0.702
HPV 16/18/33/35/52/56	0.702
HPV 16/52/53/58	0.700

[1]AUC = the area under the receiver operating characteristic (ROC) curve. The HR-HPVs are listed in descending order according to AUC values.
